# Supplementary material for: Ant venoms contain vertebrate-selective pain-causing sodium channel toxins
Source: Nat Commun. 2023 May 23;14:2977. doi: 10.1038/s41467-023-38839-1 (PMC10206162; doi:10.1038/s41467-023-38839-1)
Supplement: Supplementary file 1 — Supplementary Information [file 41467_2023_38839_MOESM1_ESM.pdf]

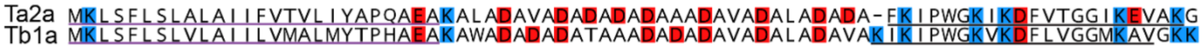

Supplementary Figure 1 | Venom peptide Ta2a from *T. africanum* is related to M-MYRTX-Tb1a (also known as bicarinalin) from venom of the ant *T. bicarinatum*. Alignment of the precursor sequence of Ta2a and M-MYRTX-Tb1a. Signal peptides and mature peptides are underlined in purple and black, respectively. Lysine/arginine and aspartate/glutamate residues are highlighted in blue and red, respectively.

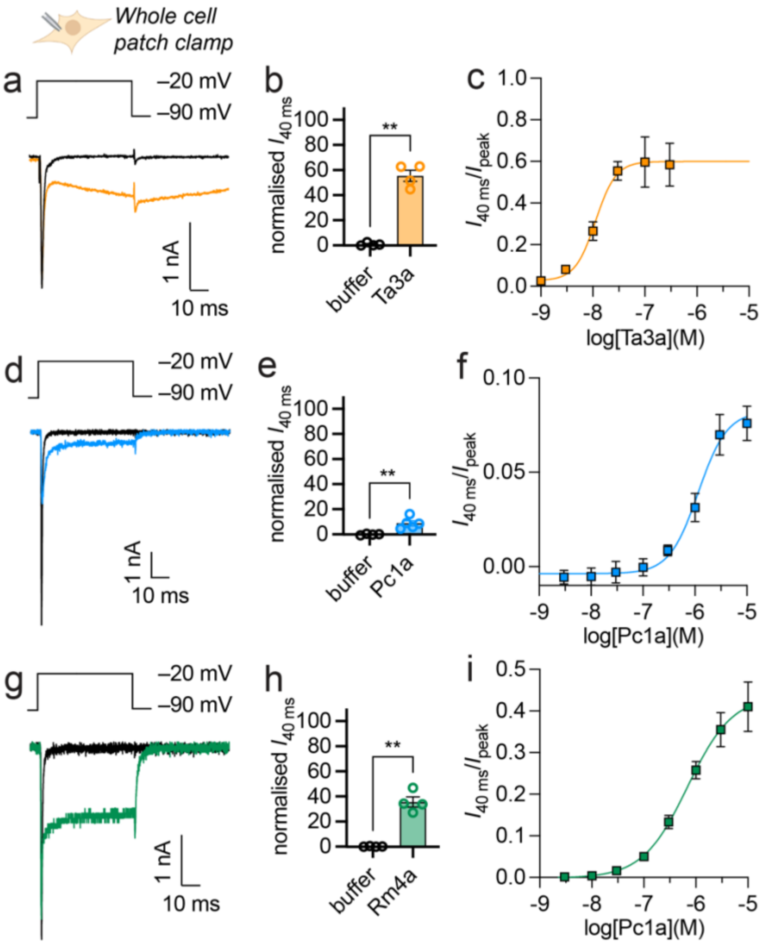

Supplementary Figure 2 | Ant venom peptides Ta3a, Pc1a and Rm4a modulate mouse Nav1.7. (a) Representative current response from a HEK293 cell expressing mNav1.7 to a step depolarization from –90 to –20 mV in the absence (black) and presence of Ta3a (30 nM, orange). Both traces shown are without leak subtraction. (b) Ta3a (30 nM) caused a sustained current of 55.4 ± 4.5 % of control peak; **, *P* = 0.0013; paired *t*-test, two-sided; *n* = 4 cells. (c) Concentration-response relationship for Ta3a modulation of mNav1.7 where response is sustained current ($I_{40\text{-ms}}$)/peak current (I_{max}) (*n* = 4 cells). (d) Representative current response from a HEK293 cell expressing mNav1.7 to a step depolarization from –90 to –20 mV in the absence (black) and presence of Pc1a (3 µM, blue). (e) Pc1a (3 µM) caused a sustained current of 8.8 ± 2.0 % of control peak; **, *P* = 0.0085; paired *t*-test, two-sided; *n* = 5 cells. (f) Concentration-response relationship for Pc1a modulation of mNav1.7 (*n* = 5 cells). (g) Representative current response from a HEK293 cell expressing mNav1.7 to a step depolarization from –90 to –20 mV in the absence (black) and presence of Rm4a (3 µM, green). (h) Rm4a (3 µM) causes a sustained current of 35.5 ± 4.1 % of control peak; **, *P* = 0.0032; paired *t*-test, two-sided; *n* = 4 cells. (i) Concentration-response relationship for Rm4a modulation of mNav1.7 (*n* = 4 cells). Data are expressed as mean ± SEM. Source data are provided as a Source Data file.

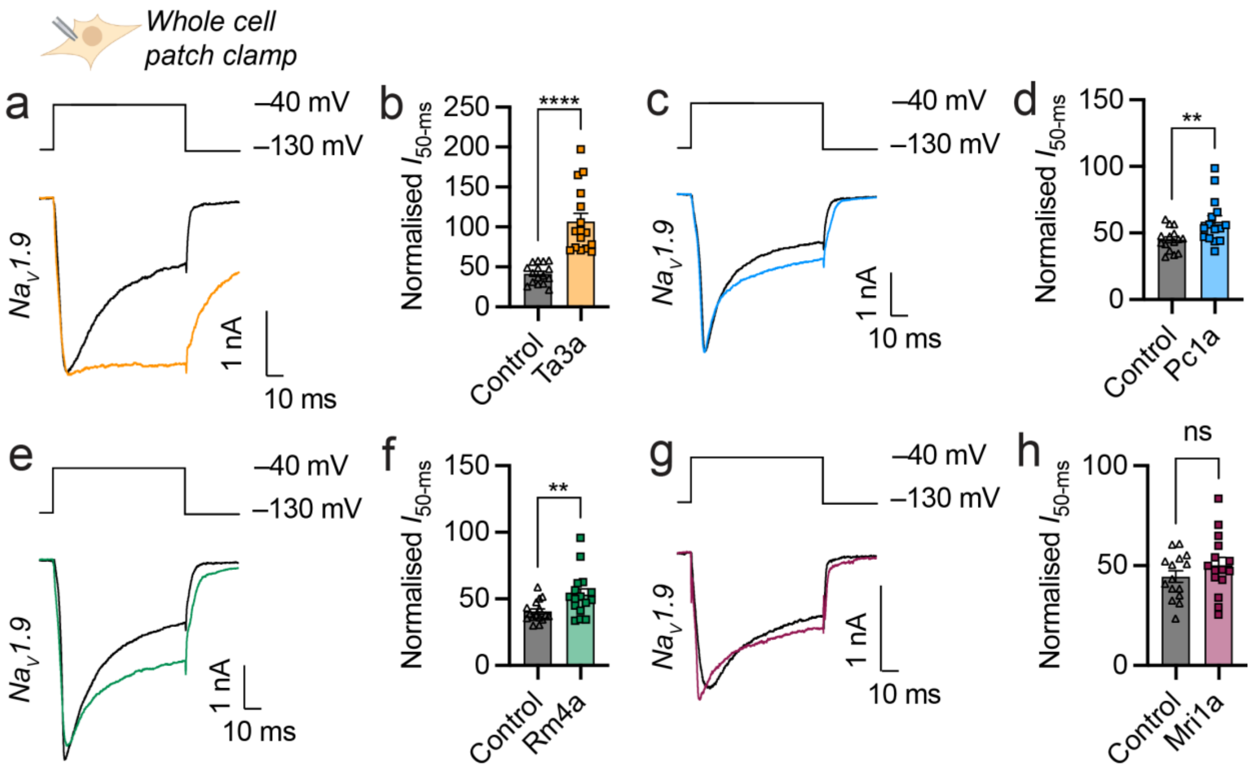

Supplementary Figure 3 | Ant venom peptides Ta3a, Pc1a and Rm4a modulate Na$_V$1.9. (a) Representative current response from a HEK293 cell expressing hNa$_V$1.9 to a step depolarization from –130 to –40 mV in the absence (black) and presence of Ta3a (1 µM, orange). (b) Na$_V$1.9 sustained current (current amplitude at 50-ms) evoked by a step depolarization from –130 to –40 mV before (control) and after treatment with Ta3a (1 µM), expressed as % of control I_{peak}. ****, $P < 0.0001$ (unpaired *t*-test; two-sided; $n = 16$ cells). (c-d) Equivalent data for Pc1a (1 µM) ($n = 15$ cells). (e-f) Equivalent data for Rm4a (1 µM). **, $P = 0.0032$ (unpaired *t*-test; two-sided; $n = 16$ cells). (g-h) Equivalent data for Mri1a (1 µM). n.s., not significant (unpaired *t*-test; two-sided; $n = 16$ cells). Data are expressed as mean ± SEM. Source data are provided as a Source Data file.

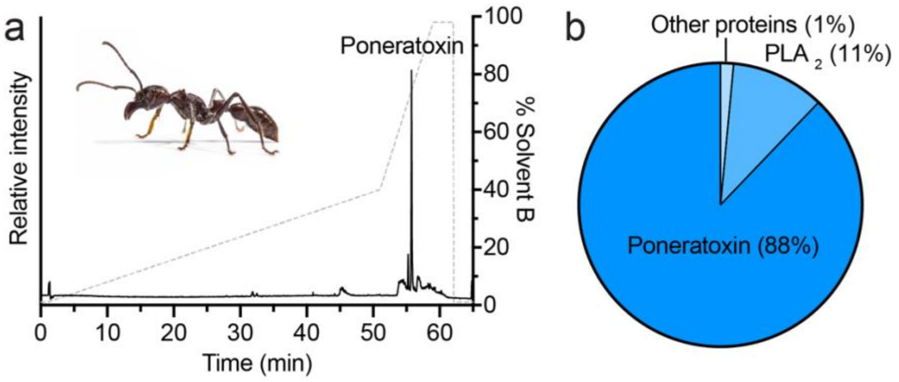

Supplementary Figure 4 | The venom of *P. clavata* is composed near-exclusively of the peptide poneratoxin. (**a**) Total ion chromatogram of *P. clavata* venom. The peak corresponding to poneratoxin is labelled. (**b**) Poneratoxin is the most highly expressed venom component-encoding transcript (defined as transcripts that encode peptides/proteins detected in the venom by LC-MS/MS) in the published venom gland transcriptome of *P. clavata*[1] where it constitutes 88% (52,149 of 59,364 transcripts per million) of venom component-encoding reads. PLA_2, phospholipase A_2.

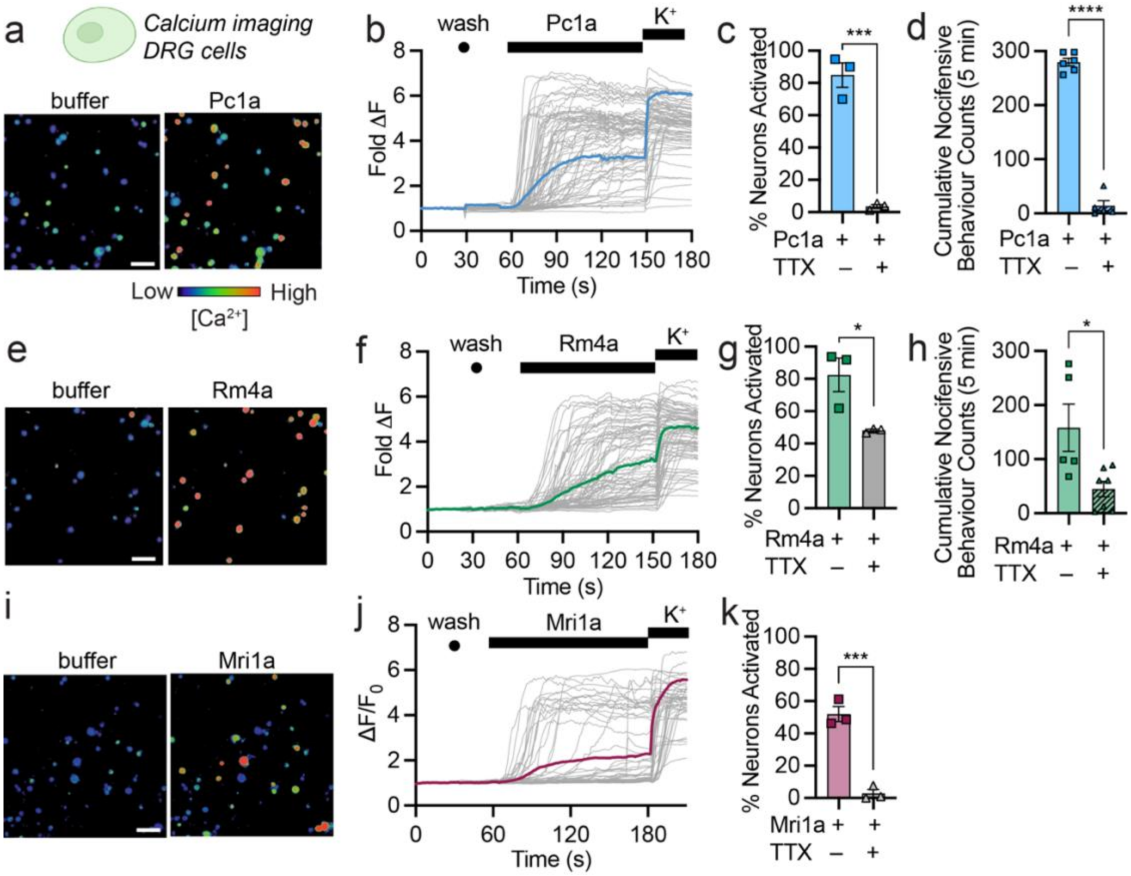

Supplementary Figure 5 | Ant venom peptide-induced activation of mouse DRG neurons and spontaneous nocifensive behaviours in mice are reduced by tetrodotoxin (TTX). (a) Representative (of 3 independent repeats) pseudocolour images illustrating $[Ca^{2+}]_i$ in DRG neurons before (buffer) and after application of Pc1a (500 nM); scale bar = 100 µm. (b) Time course of individual DRG neuron responses to Pc1a (of 3 independent repeats). Each trace represents an individual neuron. The blue trace represents the average response; K^+, 30 mM KCl (positive control). (c) Percentage of DRG neurons activated by Pc1a (500 nM) in the absence or presence of TTX (1 µM). ***, $P = 0.0004$ (unpaired *t*-test; two-sided; $n = 3$ independent experiments). (d) Pc1a (60 pmol)-induced spontaneous nocifensive behaviours with or without co-injection of 2 µM TTX. ****, $P < 0.0001$ (unpaired *t*-test; two-sided; $n = 6$ mice per group). (e) Representative (of 3 independent repeats) pseudocolour images illustrating $[Ca^{2+}]_i$ in DRG neurons before (buffer) and after application of Rm4a (500 nM). (f) Time course of individual DRG neuron responses to Rm4a (representative of 3 independent repeats). (g) Percentage of DRG neurons activated by Rm4a (500 nM) in the absence or presence of TTX (1 µM). ***, $P = 0.00297$ (unpaired *t*-test; two-sided; $n = 3$ independent experiments). (h) Cumulative spontaneous nocifensive behaviours in mice after intraplantar injection of saline or TTX (2 µM), 30 min after injection of Rm4a (60 pmol). *, $P = 0.0175$ (unpaired *t*-test; two-sided; $n = 5$ mice per group). (i) Representative (of 3 independent repeats) pseudocolour images illustrating $[Ca^{2+}]_i$ in DRG neurons before (buffer) and after application of Mri1a (1 µM). (j) Time course of individual DRG neuron responses to Mri1a (representative of 3 independent repeats). (k) Percentage of DRG neurons activated by Mri1a (1 µM) in the absence or presence of TTX (1 µM). ***, $P < 0.0007$ (unpaired *t*-test; two-sided; $n = 3$ independent experiments). Data are expressed as mean ± SEM. Source data are provided as a Source Data file.

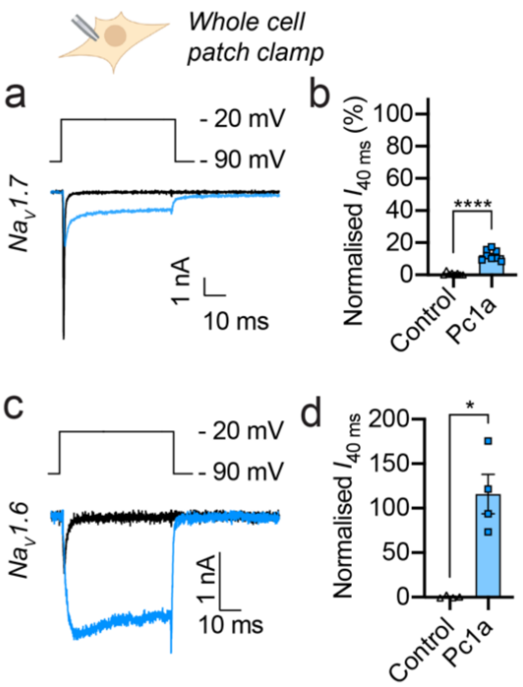

Supplementary Figure 6 | Poneratoxin (Pc1a) modulates hNaV1.7 and hNaV1.6. (a) Representative hNaV1.7 current response to a step depolarisation from –90 to –20 mV in the absence (black) and presence of 3 µM Pc1a (blue). (b) hNaV1.7 sustained current (current amplitude at 40-ms) evoked by a step depolarisation from –90 to –20 mV before (control) and after treatment with Pc1a (3 µM), expressed as % of control I_{peak} (n = 6 cells). (c) Representative hNaV1.6 current response to a step depolarisation from –90 to –20 mV in the absence (black) and presence of 3 µM Pc1a (blue). (d) hNaV1.6 sustained current (current amplitude at 40-ms) evoked by a step depolarisation from –90 to –20 mV before (control) and after treatment with Pc1a (3 µM), expressed as % of control I_{peak} (n = 4 cells). Data are expressed as mean ± SEM; ****, $P < 0.0001$; *, $P < 0.05$ (unpaired *t*-test; two-sided). Source data are provided as a Source Data file.

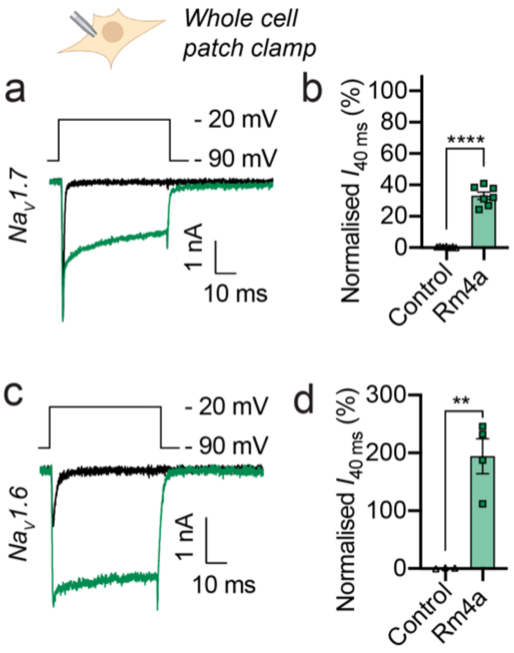

Supplementary Figure 7 | Rm4a modulates hNa$_V$1.7 and hNa$_V$1.6. (a) Representative hNa$_V$1.7 current response to a step depolarisation from –90 to –20 mV in the absence (black) and presence of 3 µM Rm4a (green). (b) hNa$_V$1.7 sustained current (current amplitude at 40-ms) evoked by a step depolarisation from –90 to –20 mV before (control) and after treatment with Rm4a (3 µM), expressed as % of control I_{peak} (n = 7 cells). (c) Representative hNa$_V$1.6 current response to a step depolarisation from –90 to –20 mV in the absence (black) and presence of 3 µM Rm4a (green). (d) hNa$_V$1.6 sustained current (current amplitude at 40-ms) evoked by a step depolarisation from –90 to –20 mV before (control) and after treatment with Rm4a (3 µM), expressed as % of control I_{peak} (n = 4 cells). Data are expressed as mean ± SEM; ****, $P < 0.0001$; **, $P < 0.01$ (unpaired t-test; two-sided). Source data are provided as a Source Data file.

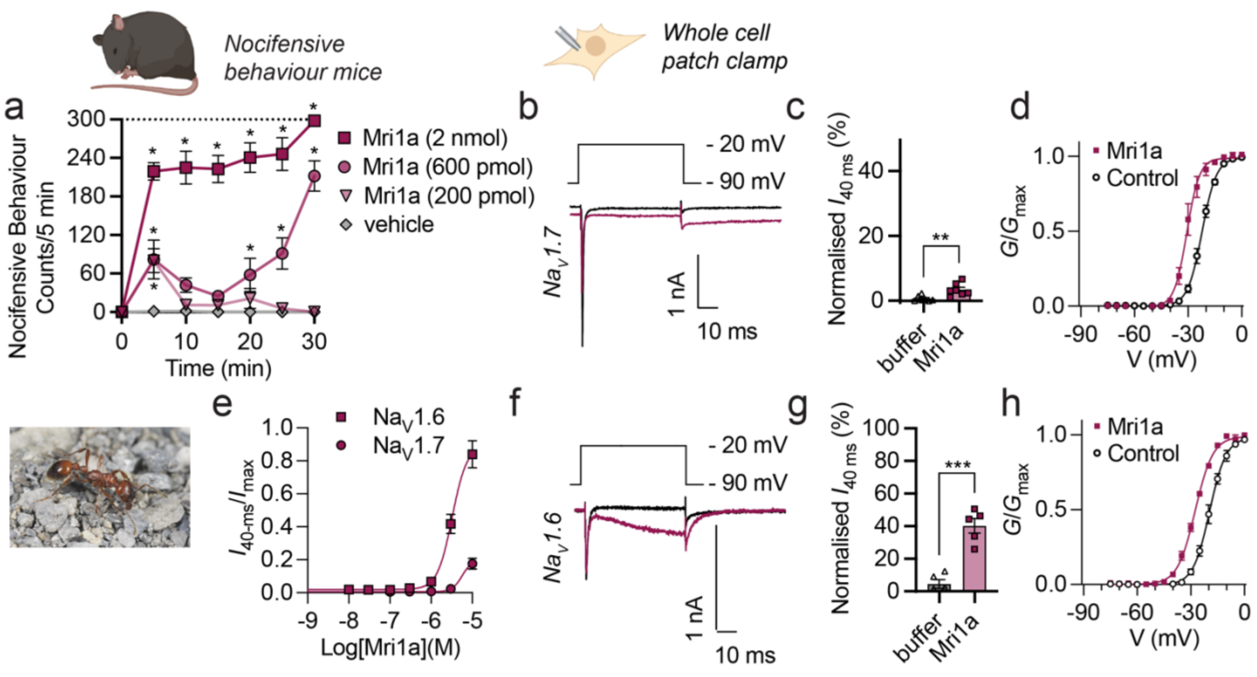

Supplementary Figure 8 | Activity of Mri1a from the venom of *M. rubida*. (a) Spontaneous nocifensive behaviours in mice following shallow intraplantar injection of Mri1a (n = 3 mice per group). *, $P < 0.05$ (two-way ANOVA with Holm-Šídák's multiple-comparisons to negative control). Inset: *M. rubida* worker (~7 mm in length). Photo credit: Thibaud Monnin. (b) Representative hNaV1.7 current response (without leak subtraction) to a step depolarisation from –90 to –20 mV in the absence (black) and presence of 3 µM Mri1a (maroon). (c) NaV1.7 sustained current (current amplitude at 40-ms) evoked by a step depolarisation from –90 to –20 mV before (control) and after treatment with Mri1a (3 µM), expressed as % of control I_{peak} **, P = 0.0061; unpaired t-test, two-sided; n = 7 cells. (d) NaV1.7 G-V curve, before (white) and after addition of 3 µM Mri1a (maroon) (n = 5 cells). (e) Concentration-response relationship for Mri1a modulation of hNaV1.6 (n = 5 cells) and hNaV1.7 (n = 7 cells), where response was [(sustained current ($I_{40\text{-ms}}$) after Mri1a treatment)/(peak current (I_{peak}) before treatment (control)]. (f) Representative hNaV1.6 current response (without leak subtraction) to a step depolarisation from –90 to –20 mV in the absence (black) and presence of 3 µM Mri1a (maroon). (g) NaV1.6 sustained current (current amplitude at 40-ms) evoked by a step depolarisation from –90 to –20 mV before (control) and after treatment with Mri1a (3 µM), expressed as % of control I_{peak}. ***, P = 0.0007; unpaired t-test, two-sided; n = 5 cells. (h) NaV1.6 G-V curve, before (white) and after addition of 3 µM Mri1a (maroon) (n = 3 cells). Data are expressed as mean ± SEM. Source data are provided as a Source Data file.

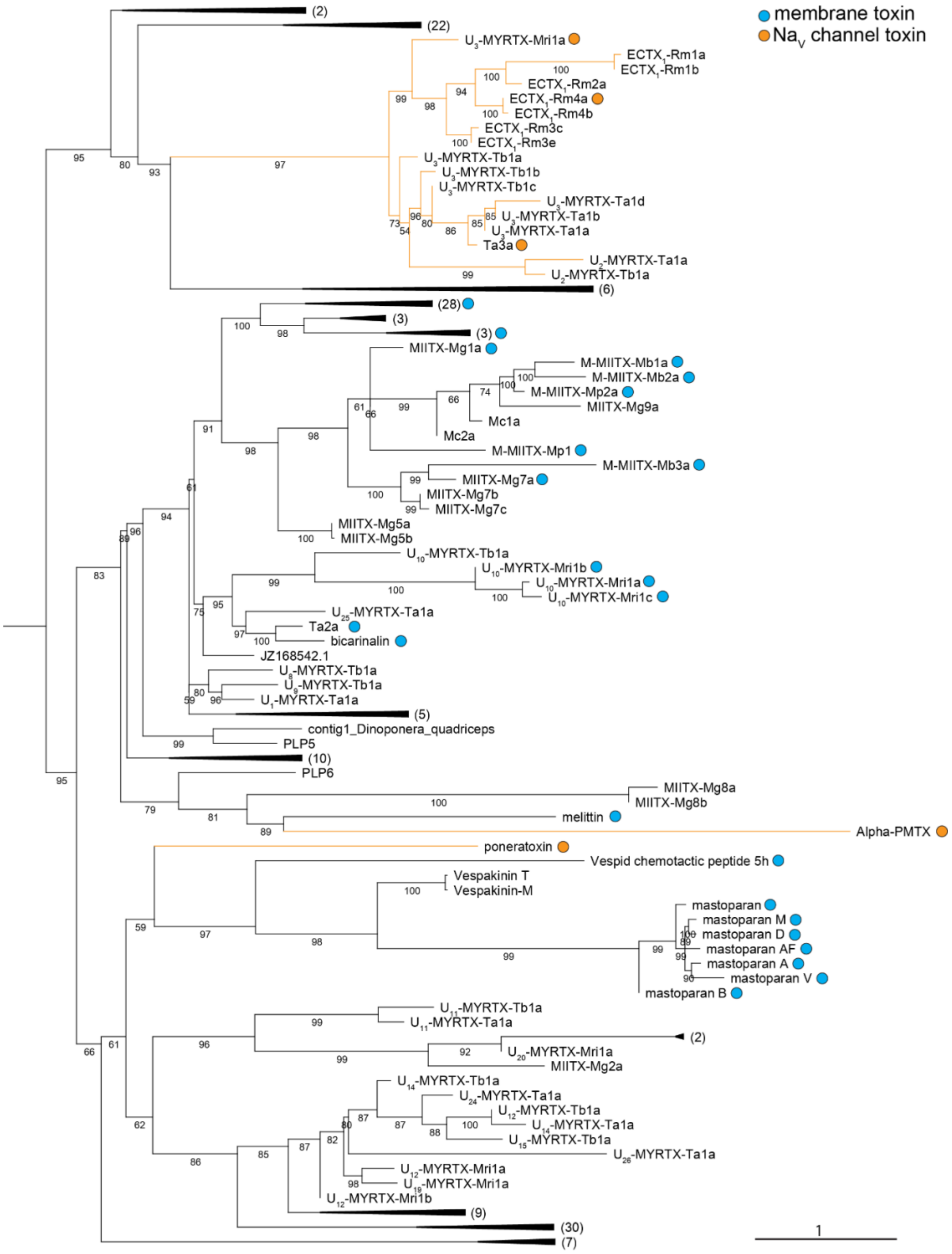

Supplementary Figure 9 | Phylogenetic reconstruction of the aculeatoxins. A maximum likelihood tree estimated using PhyML from an alignment of aculeatoxin precursor sequences. Branches representing hymenopteran venom Na_V channel toxins are coloured orange. Bootstrap values are indicated under each node.

Supplementary Table 1. Potency (µM) of ant venom Na_V channel toxins at Na_V channel subtypes.

µM	Primary structure	$hNa_V1.6$	$hNa_V1.7$	$mNa_V1.7$	$hNa_V1.8$
**Ta3a**	LAPIFALLLLSGLFSLPALQHYIEKNYIN*	0.025 ± 0.002	0.030 ± 0.009	0.018 ± 0.005	0.33 ± 0.06
**Pc1a**	FLPLLILGSLLMTPPVIQAIHDAQR*	0.097 ± 0.010	2.3 ± 0.4	1.8 ± 0.2	>10
**Rm4a**	FPPLLLLAGLFSLPALQHYIETKWIN*	0.20 ± 0.02	1.9 ± 0.4	1.2 ± 0.2	8.4 ± 1.0
**Mri1a**	GLPLLALLMTLPFIQHAITN*	3.3 ± 0.4	>10	N.D.	N.D.

*, C-terminal amidation; N.D., not determined.

Supplementary References:

1 Aili, S. R. *et al.* An Integrated Proteomic and Transcriptomic Analysis Reveals the Venom Complexity of the Bullet Ant *Paraponera clavata*. *Toxins* **12**, (2020).
