# Supplementary material for: Ant venoms contain vertebrate-selective pain-causing sodium channel toxins
Source: Nat Commun. 2023 May 23;14:2977. doi: 10.1038/s41467-023-38839-1 (PMC10206162; doi:10.1038/s41467-023-38839-1)
Supplement: Supplementary file 3 — Reporting Summary [file 41467_2023_38839_MOESM3_ESM.pdf]

nature portfolio

Corresponding author(s): Samuel Robinson, Irina Vetter

Last updated by author(s): Apr 28, 2023

Reporting Summary

Nature Portfolio wishes to improve the reproducibility of the work that we publish. This form provides structure for consistency and transparency in reporting. For further information on Nature Portfolio policies, see our Editorial Policies and the Editorial Policy Checklist.

Statistics

For all statistical analyses, confirm that the following items are present in the figure legend, table legend, main text, or Methods section.

n/a	Confirmed	
☐	☒	The exact sample size (*n*) for each experimental group/condition, given as a discrete number and unit of measurement
☒	☐	A statement on whether measurements were taken from distinct samples or whether the same sample was measured repeatedly
☐	☒	The statistical test(s) used AND whether they are one- or two-sided<br>*Only common tests should be described solely by name; describe more complex techniques in the Methods section.*
☒	☐	A description of all covariates tested
☐	☒	A description of any assumptions or corrections, such as tests of normality and adjustment for multiple comparisons
☐	☒	A full description of the statistical parameters including central tendency (e.g. means) or other basic estimates (e.g. regression coefficient) AND variation (e.g. standard deviation) or associated estimates of uncertainty (e.g. confidence intervals)
☐	☒	For null hypothesis testing, the test statistic (e.g. *F*, *t*, *r*) with confidence intervals, effect sizes, degrees of freedom and *P* value noted<br>*Give P values as exact values whenever suitable.*
☒	☐	For Bayesian analysis, information on the choice of priors and Markov chain Monte Carlo settings
☒	☐	For hierarchical and complex designs, identification of the appropriate level for tests and full reporting of outcomes
☒	☐	Estimates of effect sizes (e.g. Cohen's *d*, Pearson's *r*), indicating how they were calculated

Our web collection on statistics for biologists contains articles on many of the points above.

Software and code

Policy information about availability of computer code

Data collection	No software was used.
Data analysis	PatchMaster v1.4; GraphPad Prism9; MAFFT v7.304b; IQ-TREE v2.0.6; ModelFinder; PAUP* v4.0a;

For manuscripts utilizing custom algorithms or software that are central to the research but not yet described in published literature, software must be made available to editors and reviewers. We strongly encourage code deposition in a community repository (e.g. GitHub). See the Nature Portfolio guidelines for submitting code & software for further information.

Data

Policy information about availability of data

All manuscripts must include a data availability statement. This statement should provide the following information, where applicable:

- Accession codes, unique identifiers, or web links for publicly available datasets
- A description of any restrictions on data availability
- For clinical datasets or third party data, please ensure that the statement adheres to our policy

The data that support the findings of this study are available within the article, Supplementary Information, or Source Data file.
Sequences of Ta3a, Ta2a, Pc1a, Rm4a and Mri1a are available in Genbank: OW518818.1, Genbank: OW518839.1, UniProt: P41736, GenBank: MW317032 and GenBank: MN765042.1, respectively.

Publicly available databases used in this study: National Centre for Biotechnology Information (NCBI) nonredundant (NR) and Transcriptome Shotgun Assembly (TSA) databases; Hymenoptera Genome Database.

Human research participants

Policy information about studies involving human research participants and Sex and Gender in Research.

Reporting on sex and gender	Not applicable
Population characteristics	Not applicable
Recruitment	Not applicable
Ethics oversight	Not applicable

Note that full information on the approval of the study protocol must also be provided in the manuscript.

Field-specific reporting

Please select the one below that is the best fit for your research. If you are not sure, read the appropriate sections before making your selection.

☒ Life sciences ☐ Behavioural & social sciences ☐ Ecological, evolutionary & environmental sciences

For a reference copy of the document with all sections, see nature.com/documents/nr-reporting-summary-flat.pdf

Life sciences study design

All studies must disclose on these points even when the disclosure is negative.

Sample size	Sample size calculations were done with an alpha of 0.05 for 80% power where appropriate.
Data exclusions	No data were excluded.
Replication	All data is presented with a minimum of 3 biological replicates. All replicates yielded similar results.
Randomization	For in vivo experiments, animals were allocated to treatments by complete randomisation with allocation concealment. Randomization was not performed in in vitro experiment with pairwise design such as electrophysiological recordings pre- and post-toxin exposure.
Blinding	Experiments involving pain behaviors, consisting of paw licks or flinches, were counted from video recordings by a blinded investigator (i.e. blinded to group allocation). Blinding was not relevant to other in vitro experiments presented as measurements were analyzed by applying the same automated criteria.

Reporting for specific materials, systems and methods

We require information from authors about some types of materials, experimental systems and methods used in many studies. Here, indicate whether each material, system or method listed is relevant to your study. If you are not sure if a list item applies to your research, read the appropriate section before selecting a response.

Materials & experimental systems

n/a	Involved in the study	
☒	☐	Antibodies
☐	☒	Eukaryotic cell lines
☒	☐	Palaeontology and archaeology
☐	☒	Animals and other organisms
☒	☐	Clinical data
☒	☐	Dual use research of concern

Methods

n/a	Involved in the study	
☒	☐	ChIP-seq
☒	☐	Flow cytometry
☒	☐	MRI-based neuroimaging

Eukaryotic cell lines

Policy information about cell lines and Sex and Gender in Research

Cell line source(s)	Human Embryonic Kidney (HEK) 293 cells (American Tissue Culture Collection, Manassas, VA, USA)<br>Stable HEK293-NaV1.6 (SB Drug Discovery, Glasgow, United Kingdom)<br>Stable HEK293-NaV1.7 (SB Drug Discovery, Glasgow, United Kingdom)<br>Stable CHO-NaV1.8 (ChanTest, Ohio, USA)<br>F11 (Sigma Aldrich Australia, European Cell Culture Collection)
Authentication	None of the cell lines used were authenticated
Mycoplasma contamination	The cell lines were not tested for mycoplasma contamination
Commonly misidentified lines<br>(See ICLAC register)	No commonly misidentified cell lines were used in this study

Animals and other research organisms

Policy information about studies involving animals; ARRIVE guidelines recommended for reporting animal research, and Sex and Gender in Research

Laboratory animals	1) 5–8 week old C57BL/6J mice (male) housed in groups of up to four per cage, maintained on a 12/12 h light-dark cycle (19-21 degrees celcius; 60-70% humidity), and fed standard rodent chow and water ad libitum.<br>2) Blowflies, Lucilia caesar; adult (1-4 d post-emergence; average mass 19 mg)
Wild animals	Adult female T. africanum (worker caste; age unspecified) used for venom collection were collected from in Cameroon. They were collected, transported and maintained (briefly) in a plastic container. Specimen were dissected for collection of venom after which remaining tissue was discarded.
Reporting on sex	For experiments involving mice and mouse tissues, the sex of mice is reported and no sex- and gender-based analyses have been performed. The applicability of the findings of this study are independent of sex or gender.
Field-collected samples	This study did not involve samples collected in the field.
Ethics oversight	Experiments involving animals were approved by The University of Queensland Animal Ethics Committee (UQ AEC approval numbers PHARM/526/18 and 2021/AE000448). Experiments involving use of mouse tissue were approved by the UQ AEC (approval TRI/IMB/093/17).

Note that full information on the approval of the study protocol must also be provided in the manuscript.
